# Supplementary material for: The alternative sigma factor σX mediates competence shut-off at the cell pole in Streptococcus pneumoniae
Source: eLife. 2020 Nov 2;9:e62907. doi: 10.7554/eLife.62907 (PMC7665891; doi:10.7554/eLife.62907)
Supplement: Supplementary file 1. — Successful cin box inactivation was confirmed in three individual cin box mutants with known transformation deficits, and each strain displayed the expected transformation deficit, showing that as expected, full cin box mutation successfully abrogated σX-mediated expression, and allowing extrapolation to the 18 cin box mutants generated. [file elife-62907-supp1.docx]

| **Recipient** | **Genotype** | **Protein roles** | **Transformation efficiency (%)** | **Expected Transformants** | **Reference** |
| --- | --- | --- | --- | --- | --- |
| R4431 | *wt* |  | 6,3 |  |  |
| R4434 | *comEAC^cinbox-^* | DNA-receptor, transformation pore | 0 | None | Campbell et al., 1998 |
| R4438 | *comGA-GG^cinbox-^* | Transformation pilus | 0,00006 | Residual | Campbell et al., 1998 |
| R4440 | *comFAC^cinbox-^* | DNA-internalizing helicase | 0,000022 | Residual | Campbell et al., 1998 |
