# Supplementary material for: The alternative sigma factor σX mediates competence shut-off at the cell pole in Streptococcus pneumoniae
Source: eLife. 2020 Nov 2;9:e62907. doi: 10.7554/eLife.62907 (PMC7665891; doi:10.7554/eLife.62907)
Supplement: Supplementary file 2. [file elife-62907-supp2.docx]

| **Strain** | **Operon** | **Notable Genes** | **Protein functions** | **Reference** | **Polar DprA foci ?** |
| --- | --- | --- | --- | --- | --- |
| R4260 | *spr0023-0027* | *radA* | Transformation and genome maintenance –  Branch migration | (Burghout et al., 2007; Marie et al., 2017) | **Yes** |
| R4432 | *spr0030-0031* |  |  |  | **Yes** |
| R4433 | *spr0126-0128* | *cibABC* | Bacteriocins | (Guiral et al., 2005) | **Yes** |
| R4441 | *spr0182-0183* |  |  |  | **Yes** |
| R4442 | *spr0690* |  |  |  | **Yes** |
| R4434 | *spr0856-0857* | *comEA* | Transformation - DNA capture | (Pestova and Morrison, 1998) | **Yes** |
|  |  | *comEC* | Transformation - DNA internalization | (Pestova and Morrison, 1998) |  |
| R4435 | *spr0881-0884* | *coiA* | Unknown | (Desai and Morrison, 2006) | **Yes** |
| R4444 | *spr0996* | *radC* | Unknown | (Attaiech et al., 2008) | **Yes** |
| R4443 | *spr1003-spr1111* |  |  |  | **Yes** |
| R4436 | *spr1144* | *dprA* | Transformation, competence shut-off | (Mirouze et al., 2013; Mortier-Barrière et al., 2007) | **Yes** |
| R4448 | *spr1334* |  |  |  | **Yes** |
| R4445 | *spr1628* | *cclA* |  |  | **Yes** |
| R4437 | *spr1724* | *ssbB* | Transformation - DNA protection | (Morrison et al., 2007) | **Yes** |
| R4446 | *spr1754-1758* | *cinA* | Unknown |  | **Yes** |
|  |  | *recA* | Recombinase | (Martin et al., 1995) |  |
|  |  | *dinF* | Unknown |  |  |
|  |  | *lytA* | Peptidoglycan hydrolase - autolysis | (Sanchez-Puelles et al., 1986) |  |
| R4447 | *spr1831* |  |  |  | **Yes** |
| R4438 | *spr1858-1964* | *comGA-GG* | Transformation pilus proteins | (Laurenceau et al., 2015, 2013) | **Yes** |
| R4439 | *spr2006* | *cbpD* | Peptidoglycan hydrolase, Fratricide | (Guiral et al., 2005) | **Yes** |
| R4440 | *spr2012-2013* | *comFA* | ATP-dependent helicase | (Diallo et al., 2017) | **Yes** |
|  |  | *comFC* | Unknown | (Diallo et al., 2017) |  |
