# Supplementary material for: The alternative sigma factor σX mediates competence shut-off at the cell pole in Streptococcus pneumoniae
Source: eLife. 2020 Nov 2;9:e62907. doi: 10.7554/eLife.62907 (PMC7665891; doi:10.7554/eLife.62907)
Supplement: Supplementary file 3. — a Antibiotic resistances: SpcR, spectinomycin; KanR, kanamycin; TrimR, trimethoprim; SmR, streptomycin; CmR, chloramphenicol; RifR, rifampicin; NovR, novobiocin; EryR, erythromycin; TetR, tetracycline; AmpR, ampicillin. b Underlined, italic bases represent restriction sites used for cloning. References cited in this file: Campbell et al., 1998; Burghout et al., 2007; Desai and Morrison, 2006; Attaiech et al., 2008; Morrison et al., 2007; Martin et al., 1995; Sanchez-Puelles et al., 1986; Laurenceau et al., 2013; Laurenceau et al., 2015; Diallo et al., 2017; Lefevre et al., 1979; Caymaris et al., 2010; Mortier-Barrière et al., 2019; Akerley et al., 1998; van Raaphorst et al., 2017; Guiral et al., 2006. [file elife-62907-supp3.docx]

| **Strain** | **Genotype^a^** | **Source/Reference** |  |
| --- | --- | --- | --- |
| R209 | *recA::cat*; Cm^R^ | (Martin et al., 1995) |  |
| R304 | *rpsL41, rif23, nov1*; Sm^R^, Rif^R^, Nov^R^ | (Mortier-Barrière et al., 1998) |  |
| R751 | *rpsL1, dprA::spc^21C^*; Sm^R^, Spc^R^ | (Mirouze et al., 2013) |  |
| R800 | Non-capsulated D39 derivative, with 8,651 bp deletion in the *cps* locus | (Lefevre et al., 1979) |  |
| R825 | *comC-luc*; Cm^R^ | (Bergé et al., 2002) |  |
| R895 | *ssbB-luc*; Cm^R^ | (Chastanet et al., 2001) |  |
| R1036 | *rpsL1, comC::kan-rpsL* | (Sung et al., 2001) |  |
| R1501 | *comC0* | (Dagkessamanskaia et al., 2004) |  |
| R1502 | *comC0, ssbB-luc*; Cm^R^ | (Dagkessamanskaia et al., 2004) |  |
| R1745 | *comD::kan^105^*; Kan^R^ | (Martin et al., 2013) |  |
| R1818 | *comC0, hexA::ermAM*; Ery^R^ | (Caymaris et al., 2010) |  |
| R2002 | *comC0, comC-luc, comX1::ery, comX2::tet*, Cm^R^, Tet^R^, Ery^R^ | (Martin et al., 2013) |  |
| R2585 | *comC0, dprA^AR^* | (Quevillon-Cheruel et al., 2012) |  |
| R2830 | *comC0, dprA^QNQ^* | (Quevillon-Cheruel et al., 2012) |  |
| R2018 | *comC0, ssbB-luc, dprA::spc^21C^*; Cm^R^, Spc^R^ | (Martin et al., 2013) |  |
| R3369 | *comC2D1* | (Weyder et al., 2018) |  |
| R3728 | *comC0,* *dprA-gfp*; Spc^R^ | This study |  |
| R3743 | *comC0, dprA-gfp, ssbB-luc*; Spc^R^, Cm^R^ | This study |  |
| R3797 | *comC2D1, comC-luc, comX1::ery, comW::spc*; Cm^R^, Ery^R^, Spc^R^ | This study |  |
| R3833 | *comC2D1, CEPlac-dprA, dprA::spc^21C^*; Kan^R^, Spc^R^ | (Johnston et al., 2018) |  |
| R3912 | *comC0, radA-gfp* | This study |  |
| R3914 | *rpsl1, gfp-comD*; Sm^R^ | This study |  |
| R3915 | *rpsl1, gfp-comD, ssbB-luc*; Sm^R^, Cm^R^ | This study |  |
| R3932 | *comC2D1, CEP_R_-comXW, ssbB-luc*; Kan^R^, Cm^R^ | (Weyder et al., 2018) |  |
| R4010 | *comC0, comE-gfp* | This study |  |
| R4011 | *comC0, ssbB-luc, ftsZ-mTurquoise* | (Mortier-Barrière et al., 2019) |  |
| R4015 | *comC0, dprA-gfp, hexA::ermAM*; Spc^R^, Ery^R^ | This study |  |
| R4016 | *comC0, comE-gfp, hexA::ermAM*; Ery^R^ | This study |  |
| R4045 | *comC0, CEP_R_-dprA-gfp*; Kan^R^ | This study |  |
| R4046 | *comC0, dprA-gfp^AR^*; Spc^R^ | This study |  |
| R4047 | *comC0, dprA-gfp^QNQ^*; Spc^R^ | This study |  |
| R4048 | *comC0, dprA-mKate2*; Spc^R^ | This study |  |
| R4060 | *comC0, CEP_R_-dprA-gfp, dprA::spc^21C^*; Kan^R^, Spc^R^ | This study |  |
| R4061 | *comC0, dprA-gfp, hexA::ermAM, recA::cat*; Spc^R^, Ery^R^, Cm^R^ | This study |  |
| R4062 | *comC0, dprA-mTurquoise*; Spc^R^ | This study |  |
| R4076 | *comC0, dprA-gfp, hexA::ermAM; CEP_R_-comXW*; Spc^R^, Ery^R^, Kan^R^ | This study |  |
| R4082 | *comC0, dprA-gfp ΔcomEC::kan*; Spc^R^, Kan^R^ | This study |  |
| R4085 | *rpsl1, gfp-comD, hexA::ermAM*; Sm^R^, Ery^R^ | This study |  |
| R4087 | *comC0, comE-gfp, ssbB-luc*; Cm^R^ | This study |  |
| R4088 | *comC0, dprA-gfp, hexA::ermAM; CEP_R_-comXW, cbpD::cat*; Spc^R^, Ery^R^, Kan^R^, Cm^R^ | This study |  |
| R4091 | *comC0, radA^cinbox-^, hexA::ermAM*; Ery^R^ | This study |  |
| R4095 | *rpsl1, gfp-comD, hexA::ermAM, dprA-mTurquoise*; Sm^R^, Ery^R^, Kan^R^ | This study |  |
| R4105 | *rpsl1, yfp-comD, hexA::ermAM, dprA-mTurquoise*; Sm^R^, Ery^R^, Spc^R^ | This study |  |
| R4107 | *dprA-gfp, hexA::ermAM; CEP_R_-comXW, cbpD::cat,* Δ*comCDE::trim*; Spc^R^, Ery^R^, Kan^R^, Cm^R^, Trim^R^ | This study |  |
| R4111 | *rpsl1, yfp-comD, hexA::ermAM, dprA-mTurquoise, comA::kan*; Sm^R^, Ery^R^, Spc^R^, Kan^R^ | This study |  |
| R4112 | *rpsl1, gfp-comD, comA::kan*; Sm^R^, Kan^R^ | This study |  |
| R4138 | *comC0, CEP_R_-dprA-gfp, comX2::tet*; Kan^R^, Tet^R^ | This study |  |
| R4140 | *comC0, CEP_R_-dprA-gfp, comX2::tet, comX1::ery, comW::spc*; Kan^R^, Tet^R^, Ery^R^, Spc^R^ | This study |  |
| R4150 | *comC0, hexA::ermAM, rpoD^A171V^*; Ery^R^ | This study |  |
| R4155 | *comC0, hexA::ermAM, rpoD^A171V^, comW::spc*; Ery^R^, Spc^R^ | This study |  |
| R4166 | *comC0, hexA::ermAM, rpoD^A171V^, comW::cat*; Ery^R^, Cm^R^ | This study |  |
| R4168 | *comC0, hexA::ermAM, rpoD^A171V^, comW::cat, dprA-gfp*; Ery^R^, Cm^R^, Spc^R^ | This study |  |
| R4171 | *comC0, comE-yfp, hexA::ermAM*; Ery^R^ | This study |  |
| R4176 | *comC0, comE-yfp, hexA::ermAM, dprA-mTurquoise*; Ery^R^, Spc^R^ | This study |  |
| R4222 | *comC0, CEP_R_-dprA-gfp, dprA::spc^21C^, ssbB-luc*; Kan^R^, Spc^R^, Cm^R^ | This study |  |
| R4260 | *comC0, dprA-gfp, hexA::ermAM, radC^cin-^*; Spc^R^, Ery^R^ | This study |  |
| R4261 | *comC0, CEP_lac_-dprA-gfp;* Kan^R^ | This study |  |
| R4262 | *comC0, CEP_lac_-dprA-gfp, dprA::spc^21C^*; Kan^R^, Spc^R^ | This study |  |
| R4265 | *comC0, radA^cinbox-^, hexA::ermAM, dprA-gfp*; Ery^R^, Spc^R^ | This study |  |
| R4402 | *comC2D1, CEPlac-dprA-gfp, dprA::spc^21C^, ssbB-luc*; Kan^R^, Spc^R^, Cm^R^ | This study |  |
| R4431 | *comC0, radA-gfp, dprA-mKate2*; Spc^R^ | This study |  |
| R4432 | *comC0, radA-gfp, dprA-mKate2, spr0031^cinbox-^*; Spc^R^ | This study |  |
| R4433 | *comC0, radA-gfp, dprA-mKate2, cibA^cinbox-^*; Spc^R^ | This study |  |
| R4434 | *comC0, radA-gfp, dprA-mKate2, comEA^cinbox-^*; Spc^R^ | This study |  |
| R4435 | *comC0, radA-gfp, dprA-mKate2, coiA^cinbox-^*; Spc^R^ | This study |  |
| R4436 | *comC0, radA-gfp, dprA-mKate2, radA^cinbox-^*; Spc^R^ | This study |  |
| R4437 | *comC0, radA-gfp, dprA-mKate2, ssbB^cinbox-^*; Spc^R^ | This study |  |
| R4438 | *comC0, radA-gfp, dprA-mKate2, comGA^cinbox-^*; Spc^R^ | This study |  |
| R4439 | *comC0, radA-gfp, dprA-mKate2, cbpD^cinbox-^*; Spc^R^ | This study |  |
| R4440 | *comC0, radA-gfp, dprA-mKate2, comFA^cinbox-^*; Spc^R^ | This study |  |
| R4441 | *comC0, radA-gfp, dprA-mKate2, spr0182^cinbox-^*; Spc^R^ | This study |  |
| R4442 | *comC0, radA-gfp, dprA-mKate2, spr0690^cinbox-^*; Spc^R^ | This study |  |
| R4443 | *comC0, radA-gfp, dprA-mKate2, spr1003^cinbox-^*; Spc^R^ | This study |  |
| R4444 | *comC0, radA-gfp, dprA-mKate2, radC^cinbox-^*; Spc^R^ | This study |  |
| R4445 | *comC0, radA-gfp, dprA-mKate2, cclA^cinbox-^*; Spc^R^ | This study |  |
| R4446 | *comC0, radA-gfp, dprA-mKate2, cinA^cinbox-^*; Spc^R^ | This study |  |
| R4447 | *comC0, radA-gfp, dprA-mKate2, spr1831^cinbox-^*; Spc^R^ | This study |  |
| R4448 | *comC0, radA-gfp, dprA-mKate2, spr1334 ^cinbox-^*; Spc^R^ | This study |  |
| R4451 | *comC0, comX1-gfp* | This study |  |
| R4461 | *comC0, comX1-gfp, comX2::tet*; Tet^R^ | This study |  |
| R4465 | *comC0, comX1-gfp, comX2::tet, ssbB-luc*; Tet^R^, Cm^R^ | This study |  |
| R4466 | *comC0, ssbB-luc, comX1::ery*; CmR, Ery^R^ | This study |  |
| R4467 | *comC0, ssbB-luc, comX2::tet*; CmR, Tet^R^ | This study |  |
| R4468 | *comC0, comX1-gfp, hexA::ermAM*; Ery^R^ | This study |  |
| R4469 | *comC0, comX1-gfp,* Δ*dprA::kan*; Kan^R^ | This study |  |
| R4471 | *comC0, comX1-gfp, ssbB-luc*; Cm^R^ | This study |  |
| R4472 | *comC0, comX1-yfp, hexA::ermAM*; Ery^R^ | This study |  |
| R4473 | *comC0, comX1-yfp, hexA::ermAM, dprA-mTurquoise*; Ery^R^, Spc^R^ | This study |  |
| R4489 | *comC0, CEP_R_-dprA-gfp, dprA::spc^21C^, CEPII_R_-comX*; Kan^R^, Spc^R^, Ery^R^ | This study |  |
| R4492 | *comC0, CEP_R_-dprA-gfp, dprA::spc^21C^, comC-luc*; Kan^R^, Spc^R^, Cm^R^ | This study |  |
| R4493 | *comC0, CEP_R_-dprA-gfp, dprA::spc^21C^, CEPII_R_-comX, comC-luc*; Kan^R^, Spc^R^, Ery^R^, Cm^R^ | This study |  |
| R4498 | *comC2D1, CEP_lac_-dprA, dprA::spc^21C^, CEPII_R_-comX*; Kan^R^, Spc^R^, Ery^R^ | This study |  |
| R4500 | *comC2D1, CEP_lac_-dprA, dprA::spc^21C^, CEPII_R_-comX, comC-luc*; Kan^R^, Spc^R^, Ery^R^, Cm^R^ | This study |  |
| R4509 | *comC2D1, CEP_lac_-dprA, dprA::spc^21C^, CEPII_R_-comX, comC-luc,* Δ*comW::trim*; Kan^R^, Spc^R^, Ery^R^, Cm^R^, Trim^R^ | This study |  |
| R4511 | *comC2D1, CEP_lac_-dprA, dprA::spc^21C^, comC-luc*; Kan^R^, Spc^R^, Cm^R^ | This study |  |
| R4513 | *comC0, comW-gfp, comW^+^* | This study |  |
| R4514 | *comC0, comX1-gfp, hexA::ermAM, dprA^AR^*; Ery^R^ | This study |  |
| R4573 | *comC0, dprA-gfp, hexA::ermAM; CEP_R_-comXW, ssbB-luc*; Spc^R^, Ery^R^, Kan^R^, Cm^R^ | This study |  |
| R4574 | *ΔcomCDE::trim*; Trim^R^ | This study |  |
| R4575 | *comC0, ΔcomW::trim*; Trim^R^ | This study |  |
|  |  |  |  |
| **Plasmid** | **Genotype** | **Source/reference** |  |
| pAO0 | pUC19 derivative with *spc* gene under control of strong pneumococcal promoter; Spc^R^ | This study |  |
| pEMcat | Plasmid carrying Cm^R^ in a minitransposon cassette for *mariner* mutagenesis; Cm^R^ | (Akerley et al., 1998) |  |
| pMB42 | pAO0-derived plasmid possessing *'dprA-gfp*; Spc^R^ | This study |  |
| pMB42-dprA^AR^ | pMB42 possessing *dprA^AR^* sequence | This study |  |
| pMB42-dprA^QNQ^ | pMB42 possessing *dprA^QNQ^* sequence | This study |  |
| pMB42-mTurquoise | pMB42 possessing *mTurquoise* sequence | This study |  |
| pMB42-mKate2 | pMB42 possessing *mKate2* sequence | This study |  |
| pCEP_R_-luc | pCEP derivative with the *luc* gene under control of P_R_; Kan^R^, Spc^R^ | (Johnston et al., 2016) |  |
| pCEP_R_-dprA-gfp | pCEP_R_-luc derivative with *luc* replaced by *dprA-gfp*; Kan^R^, Spc^R^ | This study |  |
| pCEP_R_-comXW | pCEP_R_-luc derivative with *luc* replaced by *comX* and *comW*; Kan^R^, Spc^R^ | This study |  |
| pCEPlac-dprA-gfp | pCEPR-dprA-gfp derivative with P_R_ replaced with P_lac_, as well as a P_syn_ promoter controlling *lacI* expression; KanR, Spc^R^ | This study |  |
| pUC57-CEPII_R_-comX | pUC57 derivative with P_R_-*comX* and Ery^R^ flanked by sequences homologous to *cpsN* and *cpsO*; Amp^R^, Ery^R^ | Genscript, USA |  |
| pMK111 | *bgaA’-P_Zn_-tet^R^-mKate2-parBp^mut^-m(sf)gfp, ’bgaA*; Amp^R^, Kan^R^ | (van Raaphorst et al., 2017) |  |
|  |  |  |  |
| **Primer** | **Sequence (5'-3')^b^** | **Source/reference** | ***Use*** |
| BM105 | ATTCGCAAGCTTTCCCTTGAACTAGTCGAAG | (Guiral et al., 2006) | *CEP_lac_-dprA-gfp* |
| CJ379 | AGTTTTGGAAGTATTTTGTCATCTA | This study | *comE-gfp* |
| CJ380 | CCTTTAGAAACCATTCCGGAACCCTCGAGCTTTTGAGATTTTTTCTCTAAAATATCTT | This study | *comE-gfp* |
| CJ381 | AAGATATTTTAGAGAAAAAATCTCAAAAGCTCGAGGGTTCCGGAATGGTTTCTAAAGG | This study | *comE-gfp* |
| CJ382 | CATTATATCAATTTCTTGCTAATTGTCAATTATTTATACAATTCATCCATACCATGTG | This study | *comE-gfp* |
| CJ383 | CACATGGTATGGATGAATTGTATAAATAATTGACAATTAGCAAGAAATTGATATAATG | This study | *comE-gfp* |
| CJ384 | CTATCAAAGAAGTAGAAGTAATAGG | This study | *comE-gfp* |
| CJ385 | TTACAAGAAAAAACATTTTAGGAGA | This study | Δ*comCDE::trim* |
| CJ410 | GCG*CCATGG*CCTAATTAGCTGAAGGAGGAATA | This study | *CEP_R_-dprA-gfp* |
| CJ411 | GCG*GGATCC*TTATTTATACAATTCATCCATACCAT | This study | *CEP_R_-dprA-gfp* |
| CJ431 | GCG*CTCGAG*TCTAGAGGATCTGGTGGAGAAGCTGCAGC | This study | *dprA-mkate2* |
| CJ432 | GCG*AAGCTT*TTAACGGTGTCCCAATTTACTAGGCAAAT | This study | *dprA-mkate2* |
| CJ454 | GCG*CTCGAG*TTATTTATACAATTCATCCATACC | This study | *dprA-mTurquouise* |
| CJ455 | GCG*CTCGAG*ATGGTTTCTAAAGGTGAAGAATTG | This study | *dprA-mTurquouise* |
| CJ471 | TCTAGAACTAGTGGATCCCCCGGGCTGCAGATAATAAAATCTCCTAAAATGTTTTTTCTT | This study | Δ*comCDE::trim* |
| CJ472 | AAGAAAAAACATTTTAGGAGATTTTATTATCTGCAGCCCGGGGGATCCACTAGTTCTAGA | This study | Δ*comCDE::trim* |
| CJ473 | TCACTTTTGAGATTTTTTCTCTAAAATATCTCAAAGCTTATCGATACCGTCGACCTCGAG | This study | Δ*comCDE::trim* |
| CJ474 | CTCGAGGTCGACGGTATCGATAAGCTTTGAGATATTTTAGAGAAAAAATCTCAAAAGTGA | This study | Δ*comCDE::trim* |
| CJ496 | ACCTTTAGAAACCATTCCGGAACCCTCGAGAAATTCAAATTCCGCAAGAACATCTTGCC | This study | *CEP_lac_-dprA-gfp* |
| CJ497 | GGCAAGATGTTCTTGCGGAATTTGAATTTCTCGAGGGTTCCGGAATGGTTTCTAAAGGT | This study | *CEP_lac_-dprA-gfp* |
| CJ498 | ACATTATCCATTAAAAATCAAACGGATCCTTATTTATACAATTCATCCATACCATGTG | This study | *CEP_lac_-dprA-gfp* |
| CJ499 | CACATGGTATGGATGAATTGTATAAATAAGGATCCGTTTGATTTTTAATGGATAATGT | This study | *CEP_lac_-dprA-gfp* |
| CJ500 | GCGCCAAGAGGAAGGATTGATCAA | This study | *CEP_lac_-dprA-gfp* |
| CJ508 | GTCGCTCATTAAAAGGTCAGTTAAT | This study | *comW-gfp* |
| CJ513 | CTAAAATATTTGTTTGTTCACGACC | This study | *comW-gfp* |
| CJ514 | CCTTTAGAAACCATTCCGGAACCCTCGAGACAAGAAATAAACCCCCGATTCATTACCA | This study | *comW-gfp* |
| CJ515 | TGGTAATGAATCGGGGGTTTATTTCTTGT*CTCGAG*GGTTCCGGAATGGTTTCTAAAGG | This study | *comW-gfp* |
| CJ516 | AATTAGTTCGGAAATTTACTAAAATTACCTTATTTATACAATTCATCCATACCATGTG | This study | *comW-gfp* |
| CJ517 | CACATGGTATGGATGAATTGTATAAATAAGGTAATTTTAGTAAATTTCCGAACTAATT | This study | *comW-gfp* |
| CJ559 | GCGGATGAAACAGGATTCGATACTTAT | This study | Δ*comW::trim* |
| CJ560 | TCTAGAACTAGTGGATCCCCCGGGCTGCAGAATCAAATACTCCTTTTCTTTTTTATAAA | This study | Δ*comW::trim* |
| CJ561 | TTTATAAAAAAGAAAAGGAGTATTTGATTCTGCAGCCCGGGGGATCCACTAGTTCTAGA | This study | Δ*comW::trim* |
| CJ562 | AATTAGTTCGGAAATTTACTAAAATTACCAAGCTTATCGATACCGTCGACCTCGAGGG | This study | Δ*comW::trim* |
| CJ563 | CCCTCGAGGTCGACGGTATCGATAAGCTTGGTAATTTTAGTAAATTTCCGAACTAATT | This study | Δ*comW::trim* |
| CJ564 | GCGCCGTCTATAGTATACCCGACCTAT | This study | Δ*comW::trim* |
| CJ643 | GCGATACATGATTGCACTTCCTAAAGA | This study | *comX1-gfp* |
| CJ644 | TCTTCACCTTTAGAAACCATTCCGGAACC*CTCGAG*ATGGGTACGGATAGTAAACT | This study | *comX1-gfp* |
| CJ645 | AGTTTACTATCCGTACCCAT*CTCGAG*GGTTCCGGAATGGTTTCTAAAGGTGAAGA | This study | *comX1-gfp* |
| CJ646 | ATTTTTTTCATTTTTTTTGCATGACTTATTATTTATACAATTCATCCATACCATGT | This study | *comX1-gfp* |
| CJ647 | ACATGGTATGGATGAATTGTATAAATAATAAGTCATGCAAAAAAAATGAAAAAAAT | This study | *comX1-gfp* |
| CJ648 | GCGGAGAGCCGCTTTCGCCACCGGTGT | This study | *comX1-gfp* |
| DDL48 | GTGATATAATAAAAAGAGAAGAAATATGACTGTACGTCAT | This study | *spr0031^cinbox-^* |
| DDL49 | CAAGTATTTTTCAAACTTTTA*GGATCC*TAATAGATAGAGCCAGAGAAT | This study | *spr0031^cinbox-^* |
| DDL50 | ATTCTCTGGCTCTATCTATTA*GGATCC*TAAAAGTTTGAAAAATACTTG | This study | *spr0031^cinbox-^* |
| DDL51 | ATTTTCGCTGAGTTTTTCAAGTAAAATAGGCTCTGTTTCT | This study | *spr0031^cinbox-^* |
| DDL52 | CCGATGGCAAAAACGCCGACTTCTTCAGGTTTAAGAGTGT | This study | *cibA^cinbox-^* |
| DDL53 | GCTCACTTTTCCTTTTCTTTA*GGATCC*TTAAAAGTGAACAAGAAAAAA | This study | *cibA^cinbox-^* |
| DDL54 | TTTTTTCTTGTTCACTTTTAA*GGATCC*TAAAGAAAAGGAAAAGTGAGC | This study | *cibA^cinbox-^* |
| DDL55 | TCACGTCCTCTTCGGTAATCCCTGCTTCTGCCAATGCCTC | This study | *cibA^cinbox-^* |
| DDL56 | CTCACACTTGCTTCAATGTTGAATGAATTAGACATAAGAG | This study | *comEA^cinbox-^* |
| DDL57 | TATTTTCTCCTCTCTTAGATA*GGATCC*TAGAGGAAGAAAAAACAGTCG | This study | *comEA^cinbox-^* |
| DDL58 | CGACTGTTTTTTCTTCCTCTA*GGATCC*TATCTAAGAGAGGAGAAAATA | This study | *comEA^cinbox-^* |
| DDL59 | AGTCAGCCATTTCAACTTTTCTTGGGTCAAGCCCAATCGC | This study | *comEA^cinbox-^* |
| DDL60 | GATAAGCGTAAGCATTCACAAAAAGACAAGAAGAAAAAAG | This study | *coiA^cinbox-^* |
| DDL61 | AATTCCCTCCTTTTCTATATA*GGATCC*TAAAAAGAAAAAAGATCAGGA | This study | *coiA^cinbox-^* |
| DDL62 | TCCTGATCTTTTTTCTTTTTA*GGATCC*TATATAGAAAAGGAGGGAATT | This study | *coiA^cinbox-^* |
| DDL63 | GCTCCAGCCAATAATTCTTCTTCACGTTGTGAAAGAACGT | This study | *coiA^cinbox-^* |
| DDL64 | TTTGTCAAGGTCTTTGAATTCTTTCTTAAACAAGCCTTGT | This study | *dprA^cinbox-^* |
| DDL65 | TTTGATTTTTTACGAACTTTA*GGATCC*TGATAGATGAGTAGAAAAAGA | This study | *dprA^cinbox-^* |
| DDL66 | TCTTTTTCTACTCATCTATCA*GGATCC*TAAAGTTCGTAAAAAATCAAA | This study | *dprA^cinbox-^* |
| DDL67 | CTCTTGGTAAAAGAAGGGAAAGTTCGCCTCTCAATTTTCTTAAATATT | This study | *dprA^cinbox-^* |
| DDL68 | CTGTGAAAGGTAACCACGGTCAAACTGCATTCCTTCTACG | This study | *ssbB^cinbox-^* |
| DDL69 | TGTAACTTTTTTTGATTTCCA*GGATCC*TGATAAGTAGGAGGAAGAAAA | This study | *ssbB^cinbox-^* |
| DDL70 | TTTTCTTCCTCCTACTTATCA*GGATCC*TGGAAATCAAAAAAAGTTACA | This study | *ssbB^cinbox-^* |
| DDL71 | ACAATGATTTTTACGTATAACAAAGAACATGTCGGTGATG | This study | *ssbB^cinbox-^* |
| DDL72 | GAAAAGACACTCATCTTAGAACTTCTTTTATAAAAAGTGA | This study | *comGA^cinbox-^* |
| DDL73 | TTTTTATTTTTCTAACTCTTA*GGATCC*TGTATAGGTGAGGAGGTAAGT | This study | *comGA^cinbox-^* |
| DDL74 | ACTTACCTCCTCACCTATACA*GGATCC*TAAGAGTTAGAAAAATAAAAA | This study | *comGA^cinbox-^* |
| DDL75 | ACTTGCCAAAGTGGTACCATTCTTGGCCACGAAGGGATTG | This study | *comGA^cinbox-^* |
| DDL76 | TGATAGCGCAAAATAAGAAAAATGTCAAGGAAACTGCTTA | This study | *cbpD^cinbox-^* |
| DDL77 | TTCTCACTTTTTCTTTTTTCAA*GGATCC*TTTTAGGTGAAGGCAATCATC | This study | *cbpD^cinbox-^* |
| DDL78 | GATGATTGCCTTCACCTAAAA*GGATCC*TTGAAAAAAGAAAAAGTGAGAA | This study | *cbpD^cinbox-^* |
| DDL79 | GTGGTGATGCCATAGATGCAACAACGGTTCGTACTATCCT | This study | *cbpD^cinbox-^* |
| DDL80 | CACTTTGTTGACAACTCTGTTTTCATACAATTTGGACAGT | This study | *comFA^cinbox-^* |
| DDL81 | TCATCTAAAGTGCTAGTTTTA*GGATCC*TTAGAAGTATGAAAGTAAATTT | This study | *comFA^cinbox-^* |
| DDL82 | AAATTTACTTTCATACTTCTAA*GGATCC*TAAAACTAGCACTTTAGATGA | This study | *comFA^cinbox-^* |
| DDL83 | CTAATCCATCTTTACCGAAAGCAGCTAGTATCTCAGCTCC | This study | *comFA^cinbox-^* |
| DDL84 | ATGGCTCCCATTTTATGGATTACTGTCCTCATTCTACTGA | This study | *spr0182^cinbox-^* |
| DDL85 | ATTCCCCTCCTCTTTCTTTCA*GGATCC*TAAAGACAAGAAAAATAGGTC | This study | *spr0182^cinbox-^* |
| DDL86 | GACCTATTTTTCTTGTCTTTA*GGATCC*TGAAAGAAAGAGGAGGGGAAT | This study | *spr0182^cinbox-^* |
| DDL87 | CCGTATTTGTCTCCTCTTTTACCAAATCAGCAAGACGATT | This study | *spr0182^cinbox-^* |
| DDL88 | GATATTTTAACCCTCTTTCCAGAGATGTTTTCTCCACTGG | This study | *spr0690^cinbox-^* |
| DDL89 | CTTTTTTAGTTTCATTAGTTA*GGATCC*TAAATCTTGTAGATTTTAGGA | This study | *spr0690^cinbox-^* |
| DDL90 | TCCTAAAATCTACAAGATTTA*GGATCC*TAACTAATGAAACTAAAAAAG | This study | *spr0690^cinbox-^* |
| DDL91 | ACAGAAGAGCAAGCTATTAAAGAATACGGTCAAGACCAAA | This study | *spr0690^cinbox-^* |
| DDL92 | AGTCGATAATGGCGATAGGAGCATCAAGATATTCAGCCAG | This study | *spr1003^cinbox-^* |
| DDL93 | ATTTTGCTTACGATATTGCTA*GGATCC*TCCACGAAGTTTAATCTTTAA | This study | *spr1003^cinbox-^* |
| DDL94 | TTAAAGATTAAACTTCGTGGA*GGATCC*TAGCAATATCGTAAGCAAAAT | This study | *spr1003^cinbox-^* |
| DDL95 | GGCCAGCAATCCCTTAGAAACCTCGTGCTTTTTTAAAAAG | This study | *spr1003^cinbox-^* |
| DDL96 | GAAAATTACCGCTCAACCAAAACCATTCTTCAAGCGGCCA | This study | *radC^cinbox-^* |
| DDL97 | AAAAATCCTCCTCACTTTATA*GGATCC*TAGAAGGATGGAAAATTAGAT | This study | *radC^cinbox-^* |
| DDL98 | ATCTAATTTTCCATCCTTCTA*GGATCC*TATAAAGTGAGGAGGATTTTT | This study | *radC^cinbox-^* |
| DDL99 | TCGTTCAGTATGTCGATTTGACAAGTGAACTCCAAACCCT | This study | *radC^cinbox-^* |
| DDL101 | ACAGCATGAAGCAGACTCAGTAAGCCTTCAAGATCGCGTA | This study | *cclA^cinbox-^* |
| DDL102 | AAAAATCAATCATACTTATCA*GGATCC*TAAAAATGGGAGAAATAGTAT | This study | *cclA^cinbox-^* |
| DDL103 | ATACTATTTCTCCCATTTTTA*GGATCC*TGATAAGTATGATTGATTTTT | This study | *cclA^cinbox-^* |
| DDL104 | TGAGGAATTGGCTCTTTATGCCAGGGAAAAATTAGGTATT | This study | *cclA^cinbox-^* |
| DDL105 | TTGCTCCGCACGTTCACCCAAACGCATGATTGATCCTTTA | This study | *cinA^cinbox-^* |
| DDL106 | CTGCGATTTTTTCAAAAAAAA*GGATCC*TGATAGGTAGGAGGAAACATG | This study | *cinA^cinbox-^* |
| DDL107 | CATGTTTCCTCCTACCTATCA*GGATCC*TTTTTTTTGAAAAAATCGCAG | This study | *cinA^cinbox-^* |
| DDL108 | AGGAGATGGAAATATTCCTCAACAAGCAAGCTAGTTCGGA | This study | *cinA^cinbox-^* |
| DDL109 | GCATCCCAGAGCTTCCCATCAATCTCGCCACTATCATCTT | This study | *rmuC^cinbox-^* |
| DDL110 | TTAAAAAAGTGTACGCTTCTT*GGATCC*TTATAGATAGGGAAGTGTCGG | This study | *rmuC^cinbox-^* |
| DDL111 | CCGACACTTCCCTATCTATAA*GGATCC*AAGAAGCGTACACTTTTTTAA | This study | *rmuC^cinbox-^* |
| DDL112 | ACACCAGGATTTTCATCCTTGGACTATGAAGTATCAAGGG | This study | *rmuC^cinbox-^* |
| DDL114 | GGAGCTCAAGCCGTCAATTGGGGAGCTTCAGGAGGGGCTA | This study | *spr1831^cinbox-^* |
| DDL115 | TTTATTTTTTTATTTTTCATA*GGATCC*TATATAGATGAAGGGGAAAGA | This study | *spr1831^cinbox-^* |
| DDL116 | TCTTTCCCCTTCATCTATATA*GGATCC*TATGAAAAATAAAAAAATAAA | This study | *spr1831^cinbox-^* |
| DDL117 | ATCGAAAAAATCTTCTGGATGCGTTTTGAACCAGTAGTCC | This study | *spr1831^cinbox-^* |
| DDL118 | TTTTCGAGTAAGTACGAAGATTGGATAACATCAAAGAAAG | This study | *spr1334^cinbox-^* |
| DDL119 | ATAAGCTATTAGCTTTCTTTA*GGATCC*TAATAGATAGAAGCATAGAAT | This study | *spr1334^cinbox-^* |
| DDL120 | ATTCTATGCTTCTATCTATTA*GGATCC*TAAAGAAAGCTAATAGCTTAT | This study | *spr1334^cinbox-^* |
| DDL121 | GTTTAATGAGTTCAAAGATACGGCCAGGAGTTCCAATCAG | This study | *spr1334^cinbox-^* |
| dprA22 | CT*GAATTC*TAGATGACTGTTATCCTTG | This study | pMB42 |
| dprA23 | TCA*CTCGAG*AAATTCAAATTCCGCAAGAACATC | This study | pMB42 |
| MB117 | AATCTCCGCTGTAGGTCACTTTCTT | (Marie et al., 2017) | *rpsL41* |
| MB120 | TTGGATTGGGTGTGCATTTGC | (Marie et al., 2017) | *rpsL41* |
| MP216 | ATGAAAATTAAAGTTGTAACAGTTGGG | This study | *gfp-comD* |
| MP217 | CAATTCTTCACCTTTAGAAACCATTACTCTTTCCCCTTATTTCATTACT | This study | *gfp-comD* |
| MP218 | GTAATGAAATAAGGGGAAAGAGTAATGGTTTCTAAAGGTGAAGAATTG | This study | *gfp-comD* |
| MP219 | TCCGGAACC*CTCGAG*TTTATACAATTCATCCATACCATG | This study | *gfp-comD* |
| MP220 | CATGGTATGGATGAATTGTATAAA*CTCGAG*GGTTCCGGAATGGATTTATTTGGATTTGGGACGG | This study | *gfp-comD* |
| MP221 | GCATAGACAATTGACTGAGCAACC | This study | *gfp-comD* |
| OMB4 | AGA*CTCGAG*GGTTCCGGAATGGTTTCTAAAGGTGAAG | (Mirouze et al., 2013) | pMB42 |
| OMB5 | TGT*AAGCTT*GGATTATTTATACAATTCATCCATACCATGTG | This study | pMB42 |
